# Two Acceleration-Layer Configuration Amendment Schemes of Redundant Robot Arms Based on Zhang Neurodynamics Equivalency

**DOI:** 10.3390/biomimetics9070435

**Published:** 2024-07-17

**Authors:** Zanyu Tang, Mingzhi Mao, Yunong Zhang, Ning Tan

**Affiliations:** 1 School of Computer Science and Engineering, Sun Yat-sen University, Guangzhou 510006, China; tangzy23@mail2.sysu.edu.cn (Z.T.); zhynong@mail.sysu.edu.cn (Y.Z.); tann5@mail.sysu.edu.cn (N.T.); 2 School of Computer Science and Engineering, Jishou University, Jishou 416000, China; 3 School of Software Engineering, Sun Yat-sen University, Zhuhai 519082, China

**Keywords:** configuration amendment, Zhang neurodynamics equivalency, inequality type, time-variant physical limits, redundant robot arms

## Abstract

Two innovative acceleration-layer configuration amendment (CA) schemes are proposed to achieve the CA of constrained redundant robot arms. Specifically, by applying the Zhang neurodynamics equivalency (ZNE) method, an acceleration-layer CA performance indicator is derived theoretically. To obtain a unified-layer inequality constraint by transforming from angle-layer and velocity-layer constraints to acceleration-layer constraints, five theorems and three corollaries are theoretically derived and rigorously proved. Then, together with the unified acceleration-layer bound constraint, an enhanced acceleration-layer CA scheme specially considering three-layer time-variant physical limits is proposed, and a simplified acceleration-layer CA scheme considering three-layer time-invariant physical limits is also proposed. The proposed CA schemes are finally formulated in the form of standard quadratic programming and are solved by a projection neurodynamics solver. Moreover, comparative simulative experiments based on a four-link planar arm and a UR3 spatial arm are performed to verify the efficacy and superiority of the proposed CA schemes. At last, physical experiments are conducted on a real Kinova Jaco2 arm to substantiate the practicability of the proposed CA schemes.

## 1. Introduction

In recent years, intelligent manufacturing has become a popular research direction, which realizes the automation, intelligence, and high efficiency of the production process [1,2]. As an important part of intelligent manufacturing, various types of robots are widely studied [3,4,5,6,7,8,9]. In the field of engineering, the redundant robot arm is always a hot spot in research because of its multiple degrees of freedom (DOF) with the capacity to complete various tasks, e.g., repetitive motions [10], avoidance barriers [11], and satisfaction of physical limits [12]. There are many control methods for redundant robot arms, including point-to-point control [13], continuous trajectory control [14], force (torque) control [15], and intelligent control [16]. Each of these control methods has its own characteristics and can be selected according to different application scenarios. In point-to-point control, the redundant robot arm needs swift and precise adjustment from the current configuration to the target configuration, which is called the configuration amendment (CA). For instance, the robot arm completes the CA before executing point tasks, such as hitting tasks. In addition, before performing a continuous or cyclic motion task, the redundant robot arm’s configuration must be changed to match the appropriate initial configuration. As a result, the CA is crucial to control research and is unavoidable in applications.

Besides the laborsome and inefficient manual operation method, some schemes have been reported through different control techniques to achieve the CA [17,18,19]. For example, in [17], the CA scheme for redundant robot arms based on Zhang neurodynamics (ZN) with no end-effector task explicitly assigned was proposed. The authors proposed a velocity-layer CA scheme and considered satisfying physical limits of joint angle and joint velocity. However, as the velocity-layer CA scheme showed, the joint-acceleration solution exceeds its constraint because the joint-acceleration constraint is not incorporated into the scheme formulation. The authors in [19] promoted the velocity-layer scheme and proposed an acceleration-layer CA scheme that satisfies joint-angle-layer, joint-velocity-layer, and joint-acceleration-layer physical limits. However, the redundant robot arm equipped with the above-mentioned conventional acceleration-layer CA scheme may exceed physical limits during tasks in some special situations. The conventional CA schemes mentioned above just considered the time-invariant physical limits. Sometimes, the redundant robot arms are faced with not only time-invariant physical limits but also time-variant physical limits [20]. Furthermore, the time-invariant physical limits of robot arms may change over time with physical wear and damage. Therefore, the effective CA scheme for redundant robot arms at the joint-acceleration layer with time-variant physical limits is worth developing and investigating.

In the past two decades, the Zhang neurodynamics equivalency (ZNE) method, which originates from ZN, was proposed and proved to efficiently handle some complicated time-variant problems [21,22,23,24,25,26]. It is always used to transform the time-variant problem into a physically mathematically equivalent problem at the derivative layer, as it is beneficial or more convenient to handle the original problem with equivalent effecting. Motivated by [25], this study investigates two acceleration-layer CA schemes for redundant robot arms by applying the ZNE method. Whether physical limits are time-variant or time-invariant, the proposed acceleration-layer CA schemes designed via the ZNE method can satisfy them simultaneously.

In this study, we optimize the joint-acceleration-layer CA scheme [19] with the new unified constraint based on theoretical derivation, and we make up for the research gaps about the CA scheme of redundant robot arms with three-layer time-variant physical limits via the ZNE method. To formulate the proposed schemes in standard quadratic programming (QP) form, the acceleration-layer performance indicator is derived by utilizing the equality-type ZNE first. Then, the theorems and corollaries about equivalent transformations of different-layer physical limits are derived by utilizing the inequality-type ZNE, and the unified acceleration-layer bound constraints (related to time-variant and time-invariant physical limits) are obtained and presented. Combined with the acceleration-layer performance indicator and the acceleration-layer unified bound constraint, an enhanced acceleration-layer CA scheme considering three-layer (i.e., angle-, velocity-, and acceleration-layer) time-variant physical limits and a simplified acceleration-layer CA scheme considering three-layer time-invariant physical limits are proposed. Each proposed acceleration-layer CA scheme is reformulated into a QP whose solution is obtained via a projection neurodynamics (PN) solver. The simulative experiments are performed on a four-link planar arm and a six-DOF spatial arm, and the experimental results validate the correctness, effectiveness, and efficacy of the proposed acceleration-layer CA schemes. In addition, comparative experimental results substantiate that the proposed acceleration-layer CA schemes are endowed with superiority with respect to usability and completeness over the acceleration-layer CA scheme in [19]. The physical experiments are performed, and the results further verify the practicability of the proposed CA schemes.

The rest is organized as follows. Section 2 presents the requirements of realizing the CA. Section 3 presents the derivations of two proposed acceleration-layer CA schemes, including the derivations of the acceleration-layer CA performance indicator and unified acceleration-layer bound constraint. Section 4 describes the proposed CA schemes in the form of QP together with a PN solver, as well as a conventional scheme. Section 5 presents the simulative and physical experimental results. Section 6 concludes this study. Notably, this study makes the following main contributions.

1. Different from assuming that all physical limits are time-invariant in previous studies about CA, time-variant and time-invariant physical limits are both considered, and the three-layer physical-limits satisfaction of redundant robot arms is realized.
2. Theorems and corollaries for CA schemes are theoretically derived and rigorously proved via the ZNE that includes the equality-type ZNE and the inequality-type ZNE, and they supplement and complete the ZNE theory.
3. An acceleration-layer performance indicator for the CA is theoretically derived by utilizing the equality-type ZNE, and a new unified acceleration-layer time-variant bound constraint formulated from time-variant physical limits is also obtained by utilizing the inequality-type ZNE. Therefore, an acceleration-layer CA scheme for redundant robot arms is proposed in the form of QP. In addition, a simplified acceleration-layer CA scheme considering time-invariant physical limits is also presented.
4. Simulative experiments compared with the conventional CA scheme are designed and performed, and the results substantiate the superiority of the proposed CA schemes. Moreover, physical experiments are also carried out. The results further verify the practicability and correctness of the proposed CA schemes.

## 2. Preliminary and Problem

The forward kinematics equality of redundant robot arms is written as Υ(t)=f(θ(t)), where $\Upsilon \left(t\right)\in R^{m}$ is the end-effector factual position with $\theta \left(t\right)\in R^{n}$ being the joint-angle vector and f(·) being a differentiable nonlinear function. Furthermore, the kinematics equality about the relationship between the derivative of the end-effector position vector $\dot{\Upsilon }\left(t\right)\in R^{m}$ and the derivative of the joint-angle vector $\dot{\theta }\left(t\right)\in R^{n}$ is written as

$$J\left(\theta \left(t\right)\right)\dot{\theta }\left(t\right)=\dot{\Upsilon }\left(t\right),$$

where $J\left(\theta \left(t\right)\right)=\partial f\left(\theta \left(t\right)\right)/\partial \theta (t)\in R^{m\times n}$ represents the Jacobian matrix. The above equation formulates the redundant robot arm operating at the velocity layer without considering physical limits. When the redundant robot arm with three-layer physical limits operates at the acceleration layer, the requirements of realizing the CA are described as

(1) $$\begin{array}{ccc} \mathrm{objective}\;: & \; & \theta \left(t\right)\to \theta_{d}, \end{array}$$

(2) $$\begin{array}{ccc} \mathrm{subject}\;\mathrm{to}: & \; & p^{-}\left(t\right)⩽\theta \left(t\right)⩽p^{+}\left(t\right), \end{array}$$

(3) $$\begin{array}{ccc} \;\;\;\;\;\;\;\;\;\;\;\;\;\;\;\;\;\; & \; & v^{-}\left(t\right)⩽\dot{\theta }\left(t\right)⩽v^{+}\left(t\right), \end{array}$$

(4) $$\begin{array}{ccc} \;\;\;\;\;\;\;\;\;\; & \; & a^{-}\left(t\right)⩽\ddot{\theta }\left(t\right)⩽a^{+}\left(t\right), \end{array}$$

where the symbol “⩽” means that each element in the left part is less than or equal to the corresponding element in the right part, $\theta \left(t\right)\in R^{n}$ denotes the time-variant joint-angle vector, and $\theta_{d}\in R^{n}$ denotes the desired joint-angle state (i.e., objective configuration) when time t=T, with T≫0 being the run time. Furthermore, $\dot{\theta }\left(t\right)\in R^{n}$ and $\ddot{\theta }\left(t\right)\in R^{n}$ denote the time-variant joint-velocity vector and joint-acceleration vector, respectively. In addition, $p^{\pm }\left(t\right)$, $v^{\pm }\left(t\right)$, and $a^{\pm }\left(t\right)$ are the time-variant upper and lower limits of θ(t), $\dot{\theta }\left(t\right)$, and $\ddot{\theta }\left(t\right)$, respectively. The problem cannot be easily solved because there are three different-layer inequality constraints, (2), (3), and (4).

## 3. Derivation for Acceleration-Layer Scheme

In this section, the procedure of analyses and derivations for the proposed acceleration-layer CA scheme is given; as well, the theorems and corollaries for the acceleration-layer CA scheme are given and proved theoretically. Specifically, the acceleration-layer performance indicator and unified bound constraint are derived theoretically to serve the QP formulation of the proposed acceleration-layer CA schemes.

### 3.1. Acceleration-Layer Performance Indicator

In this subsection, enlightened by the research of [19,27], we use time-variant decision vector $\ddot{\theta }\left(t\right)$ in the performance indicator to achieve the acceleration-layer CA scheme for redundant robot arms. The acceleration-layer performance indicator can be derived and explained by the following Lemma 1 [28].

**Lemma** **1.** *Suppose that the time-variant vector x(t) is continuously differentiable. With adequately large-positive design parameter λ≫0 and time t≫0,*

(5) $$\dot{x}\left(t\right)+\lambda x\left(t\right)=0$$

*is physically mathematically equivalent to x(t)=0.*

**Proof.**  The proof can be obtained by consulting that of Theorem 1 in [28]. □

Note that Lemma 1 presents a ZNE between two propositions (i.e., $\dot{x}\left(t\right)+\lambda x\left(t\right)=0$ and x(t)=0 with $0\in R^{n}$ denoting a zero vector) under certain conditions with $0\in R^{n}$ being a zero vector. This type of ZNE is called an equality-type ZNE due to the fact that the related propositions are in the form of equality and are physically mathematically equivalent to each other in the sense of ZN.

**Remark** **1.** *Lemma 1 describes the equality-type ZNE, and it originates from ZN design formula $\dot{x}\left(t\right)=-\lambda x\left(t\right)$ with x(t) being the error function. When x(0) denotes the initial error function, x(t)=x(0)exp(−λt) asymptotically approaches zero as time goes on, i.e., asymptotic behavior [29]. Meanwhile, it also exponentially approaches zero. Specifically, when x(0)=1 and time t=38/λ s, x(t) is equal to $3.3139\times 10^{-17}$, which is smaller than 64-bit floating-point precision $2^{-52}$. In practice, the error can be considered to be zero.*

**Theorem** **1.** *Suppose that time-variant vector $\theta \left(t\right)\in R^{n}$ is the joint-angle vector and that $\dot{\theta }\left(t\right)$ and $\ddot{\theta }\left(t\right)$ denote the order-1 and order-2 time derivatives of the vector θ(t), respectively. With λ denoting the large-positive design parameter, requirement (1) is transformed into the acceleration-layer performance indicator for the CA of redundant robot arms with equivalent effecting, which is formulated as*

(6) $$\frac{1}{2}{\ddot{\theta }}^{T}\left(t\right)\ddot{\theta }\left(t\right)+q^{T}\left(t\right)\ddot{\theta }\left(t\right),$$

*where the variable vector $q(t)=2\lambda \dot{\theta }\left(t\right)+\lambda^{2}\left(\theta \left(t\right)-\theta_{d}\right)\in R^{n}$.*

**Proof.**  To meet requirement (1) of the CA, dynamically minimizing $\theta \left(t\right)-\theta_{d}$ is a good choice. The ideal outcome is $\theta \left(t\right)-\theta_{d}\to 0$ as t→T. Therefore, we define two error functions and utilize the ZN formula two times to procure the acceleration-layer performance indicator that has equivalent effecting with requirement (1).

*Step 1*: By defining an error function $e_{1}\left(t\right)=\theta \left(t\right)-\theta_{d}$ and utilizing the ZN formula ${\dot{e}}_{1}\left(t\right)=-\lambda_{1}e_{1}\left(t\right)$ with $\lambda_{1}$ being the large-positive design parameter, the equality $\dot{\theta }\left(t\right)=-\lambda_{1}\left(\theta \left(t\right)-\theta_{d}\right)$ is obtained, which is rearranged as $\dot{\theta }\left(t\right)+\lambda_{1}\left(\theta \left(t\right)-\theta_{d}\right)=0$. According to the equality-type ZNE (i.e., Lemma 1), $\dot{\theta }\left(t\right)+\lambda_{1}\left(\theta \left(t\right)-\theta_{d}\right)=0$ is physically mathematically equivalent to $\theta \left(t\right)-\theta_{d}=0$ as design parameter $\lambda_{1}\gg 0$ and time t≫0.

*Step 2*: To further force $\dot{\theta }\left(t\right)+\lambda_{1}\left(\theta \left(t\right)-\theta_{d}\right)\to 0$, we define another error function $e_{2}\left(t\right)=\dot{\theta }\left(t\right)+\lambda_{1}\left(\theta \left(t\right)-\theta_{d}\right)$. By utilizing the ZN formula again, one obtains $\ddot{\theta }\left(t\right)+\lambda_{1}\dot{\theta }\left(t\right)=-\lambda_{2}\left(\dot{\theta }\left(t\right)+\lambda_{1}\left(\theta \left(t\right)-\theta_{d}\right)\right)$ with $\lambda_{2}$ being the large-positive design parameter, which is rearranged as $\ddot{\theta }\left(t\right)+\left(\lambda_{1}+\lambda_{2}\right)\dot{\theta }\left(t\right)+\lambda_{1}\lambda_{2}\left(\theta \left(t\right)-\theta_{d}\right)=0$. According to the equality-type ZNE, $\ddot{\theta }\left(t\right)+\left(\lambda_{1}+\lambda_{2}\right)\dot{\theta }\left(t\right)+\lambda_{1}\lambda_{2}\left(\theta \left(t\right)-\theta_{d}\right)=0$ is physically mathematically equivalent to $\dot{\theta }\left(t\right)+\lambda_{1}\left(\theta \left(t\right)-\theta_{d}\right)=0$, and it is also physically mathematically equivalent to $\theta \left(t\right)-\theta_{d}=0$ as design parameters $\lambda_{1},\lambda_{2}\gg 0$ and time t≫0.

For simplicity and clarity, parameters $\lambda_{1}$ and $\lambda_{2}$ are set to the same value λ, and then one obtains

(7) $$\ddot{\theta }\left(t\right)+q(t)=0$$

with $q(t)=2\lambda \dot{\theta }\left(t\right)+\lambda^{2}\left(\theta \left(t\right)-\theta_{d}\right)$. The performance indicator $\ddot{\theta }\left(t\right)+q\left(t\right)$ is physically mathematically equivalent to $\theta \left(t\right)-\theta_{d}$ as design parameter λ≫0 and time t≫0. To realize the CA of redundant robot arms at the acceleration layer, we minimize the value of $\ddot{\theta }\left(t\right)+q\left(t\right)$ instead of the value of $\theta \left(t\right)-\theta_{d}$, and thus we use the following performance indicator: ${\ddot{\theta }}^{T}\left(t\right)\ddot{\theta }\left(t\right)/2+q^{T}\left(t\right)\ddot{\theta }\left(t\right).$ Thus, the proof ends. □

**Remark** **2.** *There are the decision variable $\ddot{\theta }\left(t\right)$ and vector q(t) in Equation (7). The vector q(t) is mainly determined by $\dot{\theta }\left(t\right)$ and θ(t). The variables $\dot{\theta }\left(t\right)$ and θ(t) can be obtained by the integrator for solving Equation (7), so they are treated as a known vector in this study. In addition, if the redundant robot arm has auxiliary sensors, the variables $\dot{\theta }\left(t\right)$ and θ(t) are also obtained by the corresponding sensors. Therefore, the vector q(t) is obtained.*

### 3.2. Unified Acceleration-Layer Constraint

In scientific research, the time-variant inequality x(t)⩽0 is often encountered, where $x\left(t\right)\in R^{n}$ is time-variant. In some cases, we directly obtain the order-1 or order-2 time derivatives of a vector x(t), while obtaining the vector x(t) requires extra computation [19], and then the inequalities related to the vector x(t) are not easy to handle. In other words, obtaining the equivalent inequality at a deeper layer may be an efficient method. The inequality-type ZNE is thus proposed in this section.

#### 3.2.1. Cross-One-Layer Inequality-Type ZNE

To equivalently transform the proposition about the vector θ(t) to the proposition about the vector $\dot{\theta }\left(t\right)$, Lemma 2 [25] is presented as follows.

**Lemma** **2.** *Suppose that vector x(t) is continuously differentiable. With design parameter κ≫0 and time t≫0,*

(8) $$\dot{x}\left(t\right)+\kappa x\left(t\right)⩽0$$

*is physically mathematically equivalent to x(t)⩽0.*

Note that Lemma 2 presents a ZNE between two propositions (i.e., $\dot{x}\left(t\right)+\lambda x\left(t\right)⩽0$ and x⩽0) under certain conditions. This type of ZNE is called inequality-type ZNE because the related propositions are in the form of an inequality, and they are physically mathematically equivalent to each other in the sense of ZN. As requirement (3) of the CA needs to be satisfied, one procures the equivalent inequality constraint at the acceleration layer by utilizing Lemma 2 (i.e., the inequality-type ZNE).

**Theorem** **2.** *Suppose that vector θ(t) is smooth enough. Its order-1 time derivative $\dot{\theta }\left(t\right)$ is continuously differentiable and has a time-variant upper limit $v^{+}\left(t\right)$ and lower limit $v^{-}\left(t\right)$. Both limits are continuously differentiable, and their order-1 time derivatives are ${\dot{v}}^{+}\left(t\right)$ and ${\dot{v}}^{-}\left(t\right)$, respectively. With design parameter $\kappa_{1}\gg 0$ and time t≫0,*

(9) $$\begin{array}{c} {\dot{v}}^{-}\left(t\right)+\kappa_{1}\left(v^{-}\left(t\right)-\dot{\theta }\left(t\right)\right)⩽\ddot{\theta }\left(t\right)⩽{\dot{v}}^{+}\left(t\right)+\kappa_{1}\left(v^{+}\left(t\right)-\dot{\theta }\left(t\right)\right) \end{array}$$

*is physically mathematically equivalent to $v^{-}\left(t\right)⩽\dot{\theta }\left(t\right)⩽v^{+}\left(t\right)$.*

**Proof.**  As the vector $\dot{\theta }\left(t\right)$ has its time-variant upper limit $v^{+}\left(t\right)$ and lower limit $v^{-}\left(t\right)$, one obtains $v^{-}\left(t\right)⩽\dot{\theta }\left(t\right)⩽v^{+}\left(t\right)$. Defining $x\left(t\right)=\dot{\theta }\left(t\right)-v^{+}\left(t\right)$ and utilizing the inequality-type ZNE, one has $\ddot{\theta }\left(t\right)-{\dot{v}}^{+}\left(t\right)+\kappa_{1}\left(\dot{\theta }\left(t\right)-v^{+}\left(t\right)\right)⩽0$, i.e., the right part of (9), which is physically mathematically equivalent to $\dot{\theta }\left(t\right)-v^{+}\left(t\right)⩽0$.

In a similar manner, one defines $x\left(t\right)=v^{-}\left(t\right)-\dot{\theta }\left(t\right)$ and utilizes the inequality-type ZNE again, and then $x\left(t\right)={\dot{v}}^{-}\left(t\right)-\dot{\theta }\left(t\right)+\kappa_{1}\left(v^{-}\left(t\right)-\dot{\theta }\left(t\right)\right)⩽0$ is obtained, through which one can readily obtain the left part of (9), which is physically mathematically equivalent to $v^{-}\left(t\right)-\dot{\theta }\left(t\right)⩽0$. By combining the above equivalent-effecting propositions, the proof ends. □

According to Theorem 2, $\ddot{\theta }\left(t\right)$ satisfies (9), which has equivalent effecting with that of $\dot{\theta }\left(t\right)$ and satisfies requirement (3). When the upper limit and lower limit are time-invariant, represented as $v^{+}$ and $v^{-}$, respectively, (9) becomes

(10) $$\kappa_{1}\left(v^{-}-\dot{\theta }\left(t\right)\right)⩽\ddot{\theta }\left(t\right)⩽\kappa_{1}\left(v^{+}-\dot{\theta }\left(t\right)\right).$$

#### 3.2.2. Cross-Two-Layer Inequality-Type ZNE

To equivalently transform the proposition about the vector θ(t) to the proposition about the vector $\ddot{\theta }\left(t\right)$, the relevant theorems and corollaries are derived as follows.

**Theorem** **3.** *Suppose that vector θ(t) is smooth enough with $\dot{\theta }\left(t\right)$ and $\ddot{\theta }\left(t\right)$ being the order-1 and order-2 time derivatives of θ(t), respectively. The vector θ(t) has a time-variant upper limit $p^{+}\left(t\right)$ whose order-1 and order-2 time derivatives are ${\dot{p}}^{+}\left(t\right)$ and ${\ddot{p}}^{+}\left(t\right)$, respectively. With the design parameter $\kappa_{2}\gg 0$ and time t≫0,*

(11) $$\ddot{\theta }\left(t\right)⩽{\ddot{p}}^{+}\left(t\right)+2\kappa_{2}\left({\dot{p}}^{+}\left(t\right)-\dot{\theta }\left(t\right)\right)+{\kappa_{2}}^{2}\left(p^{+}\left(t\right)-\theta \left(t\right)\right)$$

*is physically mathematically equivalent to $\theta \left(t\right)⩽p^{+}\left(t\right)$.*

**Proof.**  Defining $x_{1}\left(t\right)=\theta \left(t\right)-p^{+}\left(t\right)⩽0$, one obtains equivalent-effecting proposition ${\dot{x}}_{1}\left(t\right)+\kappa_{2}x_{1}\left(t\right)=\dot{\theta }\left(t\right)-{\dot{p}}^{+}\left(t\right)+\kappa_{2}\left(\theta \left(t\right)-p^{+}\left(t\right)\right)⩽0$ that is physically mathematically equivalent to $\theta \left(t\right)-p^{+}\left(t\right)⩽0$ by utilizing the inequality-type ZNE.

Next, defining $x_{2}\left(t\right)=\dot{\theta }\left(t\right)-{\dot{p}}^{+}\left(t\right)+\kappa_{2}\left(\theta \left(t\right)-p^{+}\left(t\right)\right)$ and utilizing the inequality-type ZNE again, one obtains equivalent-effecting proposition ${\dot{x}}_{2}\left(t\right)+\kappa_{2}x_{2}\left(t\right)=$ $\ddot{\theta }\left(t\right)-{\ddot{p}}^{+}\left(t\right)+\kappa_{2}\left(\dot{\theta }\left(t\right)-{\dot{p}}^{+}\left(t\right)\right)+\kappa_{2}\left(\dot{\theta }\left(t\right)-{\dot{p}}^{+}\left(t\right)+\kappa_{2}\left(\theta \left(t\right)-p^{+}\left(t\right)\right)\right)⩽0$, i.e., (11). This proposition is physically mathematically equivalent to $\dot{\theta }\left(t\right)-{\dot{p}}^{+}\left(t\right)+\kappa_{2}\left(\theta \left(t\right)-p^{+}\left(t\right)\right)⩽0$, which is also exactly physically mathematically equivalent to $\theta \left(t\right)-p^{+}\left(t\right)⩽0$. The proof ends. □

When the upper limit $p^{+}\left(t\right)$ is time-invariant, represented as $p^{+}$, (11) becomes

(12) $$\ddot{\theta }\left(t\right)⩽-2\kappa_{2}\dot{\theta }\left(t\right)+{\kappa_{2}}^{2}\left(p^{+}-\theta \left(t\right)\right).$$

**Theorem** **4.** *Suppose that vector θ(t) is smooth enough with $\dot{\theta }\left(t\right)$ and $\ddot{\theta }\left(t\right)$ being the order-1 and order-2 time derivatives of θ(t), respectively. The vector θ(t) has time-variant lower limit $p^{-}\left(t\right)$ whose order-1 and order-2 time derivatives are ${\dot{p}}^{-}\left(t\right)$ and ${\ddot{p}}^{-}\left(t\right)$, respectively. With the design parameter $\kappa_{2}\gg 0$ and time t≫0,*

(13) $$\ddot{\theta }\left(t\right)⩾{\ddot{p}}^{-}\left(t\right)+2\kappa_{2}\left({\dot{p}}^{-}\left(t\right)-\dot{\theta }\left(t\right)\right)+{\kappa_{2}}^{2}\left(p^{-}\left(t\right)-\theta \left(t\right)\right)$$

*is physically mathematically equivalent to $\theta \left(t\right)⩾p^{-}\left(t\right)$.*

**Proof.**  Similar to Theorem 3, this theorem can be proved by utilizing the inequality-type ZNE twice. The proof ends. □

When the lower limit $p^{-}\left(t\right)$ is time-invariant, represented as $p^{-}$, (13) becomes

(14) $$\ddot{\theta }\left(t\right)⩾-2\kappa_{2}\dot{\theta }\left(t\right)+{\kappa_{2}}^{2}\left(p^{-}-\theta \left(t\right)\right).$$

**Corollary** **1.** *Suppose that vector θ(t) has its time-variant and smooth-enough upper limit $p^{+}\left(t\right)$ and lower limit $p^{-}\left(t\right)$. With design parameter $\kappa_{2}\gg 0$ and time t≫0,*

(15) $$\begin{array}{cc} {\ddot{p}}^{-}\left(t\right)+2\kappa_{2}\left({\dot{p}}^{-}\left(t\right)-\dot{\theta }\left(t\right)\right)+ & {\kappa_{2}}^{2}\left(p^{-}\left(t\right)-\theta \left(t\right)\right)⩽\ddot{\theta }\left(t\right)\\ \; & ⩽{\ddot{p}}^{+}\left(t\right)+2\kappa_{2}\left({\dot{p}}^{+}\left(t\right)-\dot{\theta }\left(t\right)\right)+{\kappa_{2}}^{2}\left(p^{+}\left(t\right)-\theta \left(t\right)\right) \end{array}$$

*is physically mathematically equivalent to $p^{-}\left(t\right)⩽\theta \left(t\right)⩽p^{+}\left(t\right)$.*

**Proof.**  It can be generalized from Theorems 3 and 4. □

**Corollary** **2.** *Suppose that vector θ(t) has its time-invariant upper limit $p^{+}$ and lower limit $p^{-}$. With design parameter $\kappa_{2}\gg 0$ and time t≫0,*

(16) $$\begin{array}{c} -2\kappa_{2}\dot{\theta }\left(t\right)+{\kappa_{2}}^{2}\left(p^{-}-\theta \left(t\right)\right)⩽\ddot{\theta }\left(t\right)⩽-2\kappa_{2}\dot{\theta }\left(t\right)+{\kappa_{2}}^{2}\left(p^{+}-\theta \left(t\right)\right) \end{array}$$

*is physically mathematically equivalent to $p^{-}⩽\theta \left(t\right)⩽p^{+}$.*

**Proof.**  It can be generalized from Corollary 1. □

#### 3.2.3. Two Unified Acceleration-Layer Constraints

Considering the two cases with time-variant and time-invariant physical limits, one obtains the corresponding two unified ZNE constraints at the acceleration layer as the following theorem and corollary.

**Theorem** **5.** *Suppose that time-variant vector θ(t) is order-2 continuously differentiable with $p^{\pm }\left(t\right)$, $v^{\pm }\left(t\right)$, and $a^{\pm }\left(t\right)$ being the time-variant upper and lower limits of θ(t), $\dot{\theta }\left(t\right)$, and $\ddot{\theta }\left(t\right)$, respectively. The upper limit $u_{1}\left(t\right)=min\left\{a^{+}\left(t\right),{\dot{v}}^{+}\left(t\right)+\kappa_{1}\left(v^{+}\left(t\right)-\dot{\theta }\left(t\right)\right),{\ddot{p}}^{+}\left(t\right)+2\kappa_{2}\left({\dot{p}}^{+}\left(t\right)-\dot{\theta }\left(t\right)\right)+{\kappa_{2}}^{2}\left(p^{+}\left(t\right)-\theta \left(t\right)\right)\right\}$ and the lower limit $l_{1}\left(t\right)=max\{a^{-}\left(t\right),{\dot{v}}^{-}\left(t\right)+\kappa_{1}(v^{-}\left(t\right)$ $-\dot{\theta }\left(t\right)),{\ddot{p}}^{-}\left(t\right)+2\kappa_{2}\left({\dot{p}}^{-}\left(t\right)-\dot{\theta }\left(t\right)\right)+{\kappa_{2}}^{2}\left(p^{-}\left(t\right)-\theta \left(t\right)\right)\}$, the unified acceleration-layer time-variant bound constraint*

(17) $$l_{1}\left(t\right)⩽\ddot{\theta }\left(t\right)⩽u_{1}\left(t\right)$$

*is physically mathematically equivalent to time-variant bound constraints (2)–(4).*

**Proof.**  It can be generalized by combining time-variant bound constraints (4), (9), and (15). The proof ends. □

**Corollary** **3.** *Suppose that time-variant vector θ(t) is order-2 continuously differentiable with $p^{\pm }$, $v^{\pm }$, and $a^{\pm }$ being the time-invariant upper and lower limits of θ(t), $\dot{\theta }\left(t\right)$, and $\ddot{\theta }\left(t\right)$, respectively. The upper limit $u_{2}\left(t\right)=min\left\{a^{+},\kappa_{1}\left(v^{+}-\dot{\theta }\left(t\right)\right),-2\kappa_{2}\dot{\theta }\left(t\right)+{\kappa_{2}}^{2}\left(p^{+}-\theta \left(t\right)\right)\right\}$ and the lower limit $l_{2}\left(t\right)=max\left\{a^{-},\kappa_{1}\left(v^{-}-\dot{\theta }\left(t\right)\right),-2\kappa_{2}\dot{\theta }\left(t\right)+{\kappa_{2}}^{2}\left(p^{-}-\theta \left(t\right)\right)\right\}$, the unified acceleration-layer time-invariant bound constraint*

(18) $$l_{2}\left(t\right)⩽\ddot{\theta }\left(t\right)⩽u_{2}\left(t\right)$$

*is physically mathematically equivalent to time-invariant bound constraints (2)–(4).*

**Proof.**  It can be generalized by combining time-invariant bound constraints (4), (10), and (16). The proof ends. □

**Figure 1:**
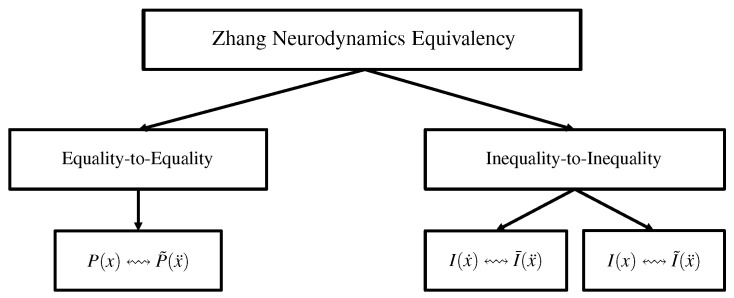
Structure plot of ZNE utilized in this study. (Note: *x* denotes time-variant variable; $\dot{x}$ denotes order-1 time derivative of *x*; $\ddot{x}$ denotes order-2 time derivative of *x*; symbol ↭ represents that propositions at both ends of the symbol have equivalent effecting; P(·) and $\tilde{P}\left(\cdot \right)$ represent propositions about performance indicators; I(·), $\bar{I}\left(\cdot \right)$, and $\tilde{I}\left(\cdot \right)$ represent propositions about inequality constraints).

In order to obtain the acceleration-layer performance index and two unified acceleration-layer constraints, we have used the ZNE multiple times. For better understanding of the ZNE applied in this study, the structure plot of corresponding the ZNE is illustrated in Figure 1.

## 4. QP Formulation and PN Solver

In this section, we first propose two acceleration-layer CA schemes. One is for the redundant robot arm with three-layer time-variant physical limits; the other is for those with three-layer time-invariant physical limits. In addition, the acceleration-layer CA scheme in [19] is also presented as a comparison.

### 4.1. Proposed and Comparative Schemes

Combined performance indicator (6) and the unified bound constraint (17), the enhanced acceleration-layer CA (EALCA) scheme for satisfying three-layer time-variant bound constraints is proposed and described as

(19) $$\begin{array}{ccc} \min.\;\;\;\;\; & \;\;\;\;\; & \frac{1}{2}{\ddot{\theta }}^{T}\left(t\right)\ddot{\theta }\left(t\right)+q_{1}^{T}\left(t\right)\ddot{\theta }\left(t\right), \end{array}$$

(20) $$\begin{array}{ccc} s.\;t.\;\;\;\;\;\; & \;\;\;\;\;\; & l_{1}\left(t\right)⩽\ddot{\theta }\left(t\right)⩽u_{1}\left(t\right), \end{array}$$

(21) $$\begin{array}{ccc} \mathrm{with}\;\;\; & q_{1}\left(t\right) & =\;2\lambda \dot{\theta }\left(t\right)+\lambda^{2}\left(\theta \left(t\right)-\theta_{d}\right), \end{array}$$

$$\begin{array}{ccc} \; & u_{1}\left(t\right) & =\;\min\{a^{+}\left(t\right),{\dot{v}}^{+}\left(t\right)\;+\;\kappa_{1}\left(v^{+}\left(t\right)\;-\;\dot{\theta }\left(t\right)\right), \end{array}$$

(22) $$\begin{array}{ccc} \;\;\;\;\;\;\;\;\; & \; & {\ddot{p}}^{+}\left(t\right)\;+\;2\kappa_{2}\left({\dot{p}}^{+}\left(t\right)\;-\;\dot{\theta }\left(t\right)\right)\;+\;{\kappa_{2}}^{2}\left(p^{+}\left(t\right)\;-\;\theta \left(t\right)\right)\}, \end{array}$$

$$\begin{array}{ccc} \; & l_{1}\left(t\right) & =\;\max\{a^{-}\left(t\right),{\dot{v}}^{-}\left(t\right)\;-\;\kappa_{1}\left(\dot{\theta }\left(t\right)\;-\;v^{-}\left(t\right)\right), \end{array}$$

(23) $$\begin{array}{ccc} \;\;\;\;\;\;\;\;\;\; & \; & {\ddot{p}}^{-}\left(t\right)\;+\;2\kappa_{2}\left({\dot{p}}^{-}\left(t\right)\;-\;\dot{\theta }\left(t\right)\right)\;+\;{\kappa_{2}}^{2}\left(p^{-}\left(t\right)\;-\;\theta \left(t\right)\right)\}. \end{array}$$

When the upper and lower limits are time-invariant, the simplified acceleration-layer CA (SALCA) scheme is obtained by combining the performance indicator (6) and the unified bound constraint (18), which is presented as follows.

(24) $$\begin{array}{ccc} \;\;\;\;\;\;\min.\;\;\;\;\; & \;\;\;\;\;\; & \frac{1}{2}{\ddot{\theta }}^{T}\left(t\right)\ddot{\theta }\left(t\right)+q_{2}^{T}\left(t\right)\ddot{\theta }\left(t\right), \end{array}$$

(25) $$\begin{array}{ccc} \;\;\;\;\;\;s.\;t.\;\;\;\;\;\; & \;\;\;\;\;\; & l_{2}\left(t\right)⩽\ddot{\theta }⩽u_{2}\left(t\right), \end{array}$$

(26) $$\begin{array}{ccc} \mathrm{with}\;\; & q_{2}\left(t\right) & =2\lambda \dot{\theta }\left(t\right)+\lambda^{2}\left(\theta \left(t\right)-\theta_{d}\right), \end{array}$$

(27) $$\begin{array}{ccc} \; & u_{2}\left(t\right) & =\min\{a^{+},\kappa_{1}\left(v^{+}-\dot{\theta }\left(t\right)\right),-2\kappa_{2}\dot{\theta }\left(t\right)+{\kappa_{2}}^{2}\left(p^{+}-\theta \left(t\right)\right)\},\;\; \end{array}$$

(28) $$\begin{array}{ccc} \; & l_{2}\left(t\right) & =\max\{a^{-},\kappa_{1}\left(v^{-}-\dot{\theta }\left(t\right)\right),-2\kappa_{2}\dot{\theta }\left(t\right)+{\kappa_{2}}^{2}\left(p^{-}-\theta \left(t\right)\right)\}.\;\; \end{array}$$

For comparison, the conventional acceleration-layer CA scheme in [19], named as the CALCA scheme, is also presented as follows.

(29) $$\begin{array}{ccc} \min.\;\;\;\;\;\; & \;\;\;\;\; & \frac{1}{2}{\ddot{\theta }}^{T}\left(t\right)\ddot{\theta }\left(t\right)+q_{3}^{T}\left(t\right)\ddot{\theta }\left(t\right), \end{array}$$

(30) $$\begin{array}{ccc} s.\;t.\;\;\;\;\;\;\; & \;\;\;\;\; & l_{3}\left(t\right)⩽\ddot{\theta }\left(t\right)⩽u_{3}\left(t\right), \end{array}$$

(31) $$\begin{array}{ccc} \mathrm{with}\;\;\; & q_{3}\left(t\right) & =\left(\beta_{1}+\beta_{2}\right)\dot{\theta }\left(t\right)+\beta_{1}\beta_{2}\left(\theta \left(t\right)-\theta_{d}\right), \end{array}$$

(32) $$\begin{array}{ccc} \;\;\;\; & u_{3}\left(t\right) & =\min\{a^{+},\nu \left(v^{+}-\dot{\theta }\left(t\right)\right),\mu \left(p^{+}-ℸ-\theta \left(t\right)\right)\},\;\; \end{array}$$

(33) $$\begin{array}{ccc} \;\;\;\;\; & l_{3}\left(t\right) & =\max\{a^{-},\nu \left(v^{-}-\dot{\theta }\left(t\right)\right),\mu \left(p^{-}-ℸ-\theta \left(t\right)\right)\}, \end{array}$$

where parameters $\beta_{1},\;\beta_{2},\;\nu$, and $\mu \in R^{+}$, and *ℸ* is a vector whose dimension is the same as vector θ(t).

### 4.2. PN Solver

The above analyses indicate that defining $y\left(t\right)=\ddot{\theta }\left(t\right)\in R^{n}$ results in the following QP reformulation of the EALCA scheme (19)–(23) and SALCA scheme (24)–(28), as well as the CALCA scheme (29)–(33):

(34) $$\begin{array}{ccc} \min. & \; & \frac{1}{2}y^{T}\left(t\right)Qy\left(t\right)+q^{T}\left(t\right)y\left(t\right), \end{array}$$

(35) $$\begin{array}{ccc} s.\;t. & \;\; & l(t)⩽y⩽u(t),\;\;\;\;\;\;\;\;\;\;\;\;\;\; \end{array}$$

where $Q=I\in R^{n\times n}$, $q\left(t\right)=q_{i}\left(t\right)$, $u=u_{i}\left(t\right)$, and $l=l_{i}\left(t\right)$ with i=1, 2, and 3. By solving QP (34) and (35) in real time, the solution y(t) can be obtained by the PN solver in this subsection. Therefore, the PN solver is developed in the following lemmas [30,31].

**Lemma** **3.** *With $\delta \in R^{+}$ adjusting the convergence rate, the PN solver for the acceleration-layer CA schemes is developed as*

(36) $$\dot{y}\left(t\right)=\delta \left(I+Q^{T}\right)\left(P_{\Omega }\left(y\left(t\right)-\left(Qy\left(t\right)-q\left(t\right)\right)\right)-y\left(t\right)\right),$$

*where coefficients $Q=I\in R^{n\times n}$ with I being the identity matrix in the manner of MATLAB [32], and $q\left(t\right)=q_{i}\left(t\right)$ with i=1,2,and3. Furthermore, $\dot{y}\left(t\right)$ is the time derivative of y(t), and $P_{\Omega }$ is a projection operator.*

**Lemma** **4.** Suppose that the optimal solution $y^{*}\left(t\right)$ to the CA exists. Starting from any initial state y(0), the variable vector y(t) of PN (36) converges to the theoretical solution $y^{*}\left(t\right)$ [24].

**Remark** **3.** *The method developed in this study depends on a special recurrent neural network to obtain solutions with minimum errors. It is suitable for both redundant and nonredundant robot arms. The kinematic optimization of redundant robot arms is realized by utilizing redundant freedom degrees. The solution space of the mathematical model of redundant robot arms is large, which makes it possible to optimize the performance indicator. Therefore, redundant robot arms can satisfy the second task of joint physical limits, singularity avoidance, and obstacle avoidance. In addition, the method developed in this study is also applicable to nonredundant robot arms in theory. The configuration of robot arms can be adjusted to the desired configuration with negligible errors if the desired one is within the range of joint-angle physical limits. Furthermore, the configuration will be adjusted to the one closest to the desired configuration if the desired one is not within the range of joint-angle physical limits. The proposed schemes in the study are currently not realized on nonredundant robot arms, while their application to nonredundant robot arms is a feasible future research direction.*

## 5. Experiments, Verification, and Comparisons

In this section, simulative experiments based on two kinds of redundant robot arms (i.e., the four-link planar arm and the UR3 spatial arm) are conducted, and the results are presented to substantiate the superior performance of the proposed acceleration-layer CA schemes.

Note that the simulative experiments are based on the fact that redundant robot arms are always constrained by physical limits. In the simulative experiments, we consider two cases. Firstly, the constraints are loose. We refer to the redundant robot arm having loose constraints in this study if the redundant robot arm is far from reaching the physical limits when carrying out a certain task. The simulative results are the same as those synthesized by the redundant robot arm without considering the constraints in [19]. Secondly, some constraints are tight. When the redundant robot arm may reach physical limits due to hardware damage or other reasons, it is referred to as having tight constraints.

### 5.1. Application to Four-Link Planar Arm

In this subsection, three groups experiments based on four-link planar arm are conducted and their results are presented.

#### 5.1.1. Experiment Group 1

In this part, the time-invariant upper and lower limits are considered first, and the SALCA scheme (24)–(28) and CALCA scheme (29)–(33) are used to perform the CA tasks based on the four-link planar arm.

This group of simulative experiments is conducted with almost the same constraints and parameters as those in [19]. Specifically, the length of each link is set to 1.2 m, the simulation duration time *T* is set to 5 s, and the joint constraints are fixed as follows.

(37) $$\begin{array}{c} p^{+}=-p^{-}={\left[1.5708,1.0472,0.7854,0.3927\right]}^{T}\;\mathrm{rad}, \end{array}$$

(38) $$\begin{array}{c} v^{+}=-v^{-}={\left[0.5000,0.5000,0.5000,0.5000\right]}^{T}\;\mathrm{rad}/s, \end{array}$$

(39) $$\begin{array}{c} a^{+}=-a^{-}={\left[1.5000,1.5000,1.5000,1.5000\right]}^{T}\;\mathrm{rad}/s^{2}. \end{array}$$

The initial configuration and desired configuration for the four-link planar arm are also, respectively, selected as ${\;\;}^{1}\theta \left(0\right)={\left[\pi /3,\pi /4,\pi /5,\pi /8\right]}^{T}$ rad and ${\;\;}^{1}\theta_{d}={\left[\pi /9,\pi /9,\pi /9,\pi /9\right]}^{T}$ rad. In addition, the other parameters are set as $\delta =10^{4}$, $\kappa_{1}=\kappa_{2}=2$, and λ=2 that keep the same values in all simulative experiments.

**Figure 2:**
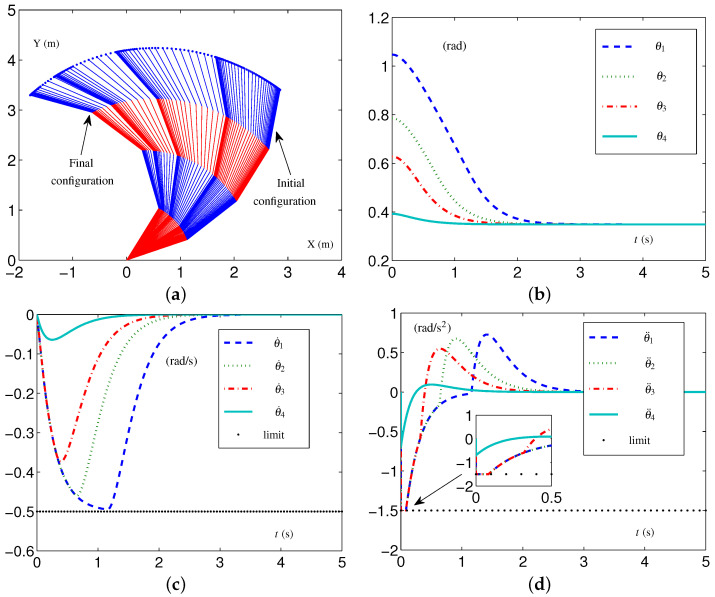
Synthesized results on four-link planar arm equipped with SALCA scheme (24)–(28) with tight velocity-layer and acceleration-layer physical limits satisfied. (**a**) Initial and final configurations. (**b**) Joint-angle trajectories. (**c**) Joint-velocity trajectories. (**d**) Joint-acceleration trajectories.

Based on these data, the specific formulations of the SALCA scheme (24)–(28) are obtained. The related simulative results are depicted in Figure 2. The four-link planar arm with initial configuration and desired configuration labeled is depicted in Figure 2a. Note that the configurations of the redundant robot arm in Figure 2a are depicted using two alternating colors (i.e., red and blue) for enhanced clarity. The use of two colors to depict the configurations is consistently applied throughout the study. The trajectories of θ(t), $\dot{\theta }\left(t\right)$, and $\ddot{\theta }\left(t\right)$ are depicted in Figure 2b through Figure 2d. From Figure 2c,d, one sees that the four-link planar arm is in the tight-constraint case regarding the velocity layer and acceleration layer, while the values of $\dot{\theta }\left(t\right)$ and $\ddot{\theta }\left(t\right)$ all keep within the allowed range and satisfy the constraints.

Furthermore, the vector ϵ is defined as $\left|\theta \left(T\right)-\theta_{d}\right|$ to indicate the effect of schemes, and its *i*th element is the value of $\left|\theta_{i}\left(T\right)-\theta_{di}\right|$ with i=1,⋯,n. The vector ϵ shows the configuration difference between the final configuration θ(T) and the desired configuration as $\theta_{d}$. The configuration difference is obtained in this simulation, i.e., $\epsilon ={\left[4.75268,0.65614,0.19725,0.01978\right]}^{T}\times 10^{-7}$ rad, which is almost the same as the value obtained by the CALCA scheme (29)–(33) in [19]. Those simulative results show that the proposed SALCA scheme (24)–(28) is effective, as is the CALCA scheme (29)–(33) under those conditions.

In addition, the EALCA scheme (19)–(23) is investigated on a four-link planar arm. The time-variant loose constraints are set as follows: each element in $p^{+}\left(t\right)$, $v^{+}\left(t\right)$, and $a^{+}\left(t\right)\in R^{4}$ is set as $1.5-0.25\sin^{2}\left(t/2\right)$ rad, $15-0.25\sin^{2}\left(t/2\right)$ rad/s, and $15-0.25\sin^{2}t$ rad/s${\;\;}^{2}$, respectively; each element in $p^{-}\left(t\right)$, $v^{-}\left(t\right)$, and $a^{-}\left(t\right)\in R^{4}$ is $-1.5+0.25\sin^{2}\left(t/2\right)$ rad, $-15+0.25\sin^{2}\left(t/2\right)$ rad/s, and $-15+0.25\sin^{2}t$ rad/s${\;\;}^{2}$, respectively. The four-link planar arm is far from reaching the time-variant physical limits during the CA task when it has the above constraints. For maintaining clarity and improving readability, we name these time-variant loose-constraint settings as TVLC settings.

**Figure 3:**
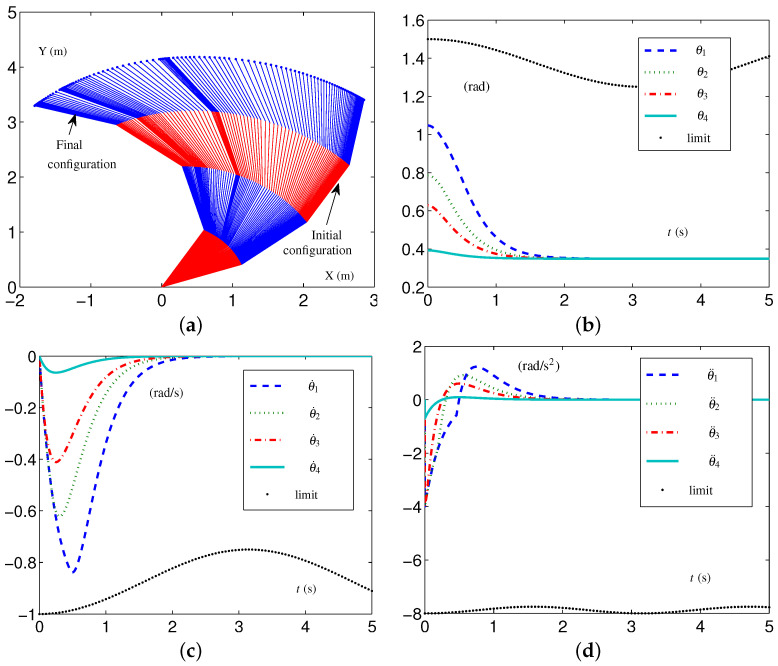
Synthesized results on four-link planar arm equipped with EALCA scheme (19)–(23) with all physical limits satisfied. (**a**) Initial and final configurations. (**b**) Joint-angle trajectories. (**c**) Joint-velocity trajectories. (**d**) Joint-acceleration trajectories.

When the four-link planar arm has TVLC settings, except for each element in $v^{-}\left(t\right)$ and $a^{-}\left(t\right)\in R^{4}$ being, respectively, changed to $-1+0.25\sin^{2}\left(t/2\right)$ rad/s and $-8+0.25\sin^{2}t$ rad/s${\;\;}^{2}$, simulative experiments are conducted to confirm the efficacy of the EALCA scheme (19)–(23). The initial configuration is ${\;\;}^{1}\theta \left(0\right)$ and the desired configuration is ${\;\;}^{1}\theta_{d}$. The related simulative results are depicted in Figure 3. From the trajectories of the variables, one obtains that the four-link planar arm equipped with the EALCA scheme (19)–(23) completes the task effectively in the tight-constraint case regarding the velocity layer and acceleration layer, and the different-layer physical limits are satisfied. The configuration difference $\epsilon ={\left[7.04179,2.52647,1.35912,0.21107\right]}^{T}\times 10^{-8}$ rad shows that the task is completed with high quality.

#### 5.1.2. Experiment Group 2

This group of simulative experiments is performed with the initial configuration being ${\;\;}^{2}\theta \left(0\right)={\;\;}^{1}\theta_{d}$ and the desired configuration ${\;\;}^{2}\theta_{d}={\;\;}^{1}\theta \left(0\right)$. That is to say, the values of the initial configuration and the desired configuration (used in Experiment Group 1) exchange, and the four-link planar arm returns to the original position from the present position after the first CA simulation.

**Figure 4:**
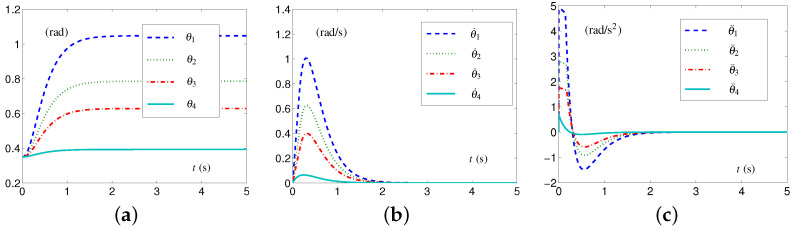
Synthesized results on four-link planar arm equipped with CALCA scheme (29)–(33) with loose limits satisfied. (**a**) Joint-angle trajectories. (**b**) Joint-velocity trajectories. (**c**) Joint-acceleration trajectories.

**Figure 5:**
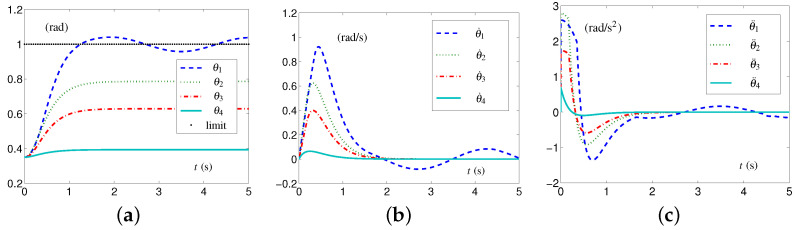
Synthesized results on four-link planar arm equipped with CALCA scheme (29)–(33) with tight constraints. (**a**) Joint-angle trajectories. (**c**) Joint-velocity trajectories. (**b**) Joint-acceleration trajectories.

The CALCA scheme (29)–(33) is investigated with the time-invariant loose constraints being fixed as follows: each element in $p^{+}$, $v^{+}$, and $a^{+}\in R^{4}$ is set as 1.5 rad, 10 rad/s, and 15 rad/s${\;\;}^{2}$, respectively; each element in $p^{-}$, $v^{-}$, and $a^{-}\in R^{4}$ is −1.5 rad, −10 rad/s, and −15 rad/s${\;\;}^{2}$, respectively. We coherently name these time-invariant loose-constraint settings as TILC settings. In addition, each parameter in vector *ℸ* is 0.001; parameters $\beta_{1}=4,\;\beta_{2}=4,\;\nu =4$, and μ=4. The related simulative results are depicted in Figure 4, which shows that the four-link planar arm equipped with the CALCA scheme (29)–(33) completed the task successfully. However, when each element in vector $p^{+}$ decreases from 1.5 rad to 1.0 rad, i.e., the four-link planar arm performs the CA task in the tight-constraint case regarding the angle layer, there is an undesirable situation. The simulative results are depicted in Figure 5. From Figure 5a, one sees that the values of $\theta_{1}$ exceed the joint-angle upper limit, and the four-link planar arm may be damaged or damaged further.

**Figure 6:**
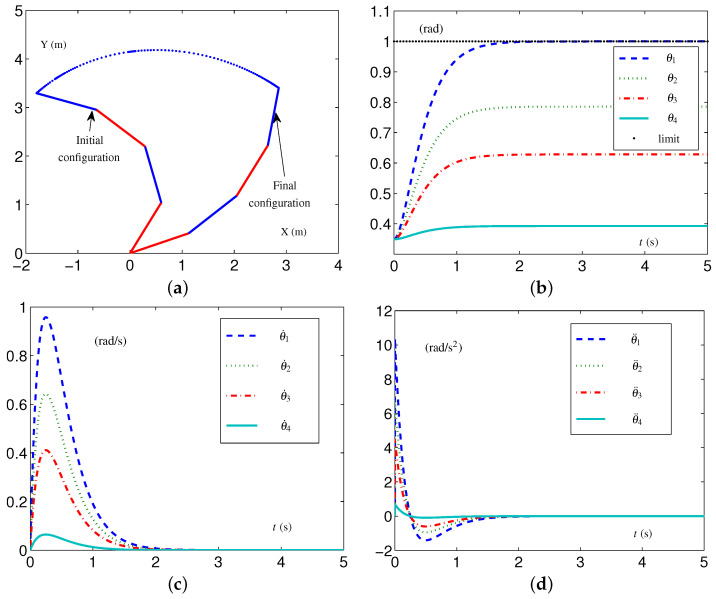
Synthesized results on four-link planar arm equipped with SALCA scheme (24)–(28) with all physical limits satisfied. (**a**) Initial and final configurations. (**b**) Joint-angle trajectories. (**c**) Joint-velocity trajectories. (**d**) Joint-acceleration trajectories.

The SALCA scheme (24)–(28) is investigated to perform the CA task with the same tight constraints as mentioned above. The simulative results are shown in Figure 6. One obtains that the values of $\theta_{1}$ do not exceed the joint-angle upper limits, as shown in Figure 6b, and the values of $\dot{\theta }$ and $\ddot{\theta }$ all keep within the allowed range, as shown in Figure 6c,d. The configuration difference $\epsilon ={\left[0.0471976,1.1661\times 10^{-8},8.03722\times 10^{-8},1.16341\times 10^{-9}\right]}^{T}$ rad is obtained. That is to say, the four-link planar arm equipped with the SALCA scheme (24)–(28) completes the CA with all physical limits satisfied.

**Figure 7:**
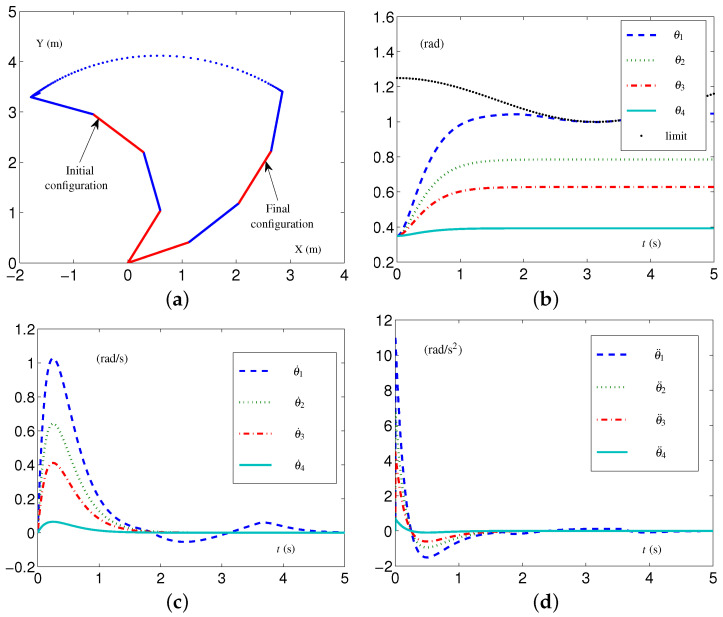
Synthesized results on four-link planar arm equipped with EALCA scheme (19)–(23) with all physical limits satisfied. (**a**) Initial and final configurations. (**b**) Joint-angle trajectories. (**c**) Joint-velocity trajectories. (**d**) Joint-acceleration trajectories.

In addition, the EALCA scheme (19)–(23) is also investigated with TVLC settings except for each element in $p^{+}\left(t\right)$ being set as $1.25-0.25\sin^{2}\left(t/2\right)$ rad. As shown in Figure 7, the four-link planar arm equipped with the EALCA scheme (19)–(23) completes the task with all physical limits satisfied. The configuration difference $\epsilon =[5.51668\times 10^{-4}$, $1.90918\times 10^{-8}$, $1.2216\times 10^{-8},1.90922\times 10^{-9}{]}^{T}$ rad is obtained. This group comparative simulation verifies that the proposed acceleration-layer CA schemes are effective and have superiority compared with the conventional CA scheme in some cases.

#### 5.1.3. Experiment Group 3

**Figure 8:**
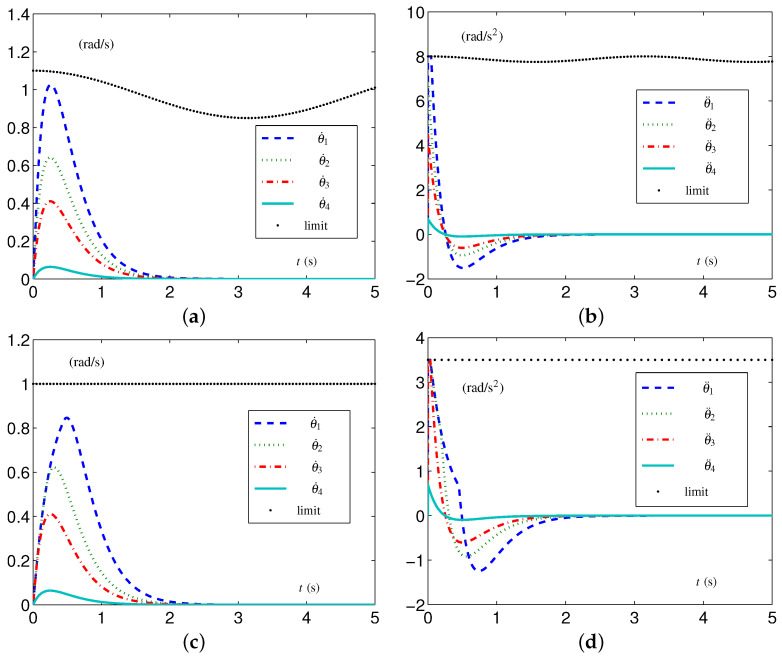
Synthesized results on four-link planar arm with tight velocity-layer and acceleration-layer physical limits satisfied. (**a**) Joint-velocity trajectories synthesized by EALCA scheme (19)–(23). (**b**) Joint-acceleration trajectories synthesized by EALCA scheme (19)–(23). (**c**) Joint-velocity trajectories synthesized by SALCA scheme (24)–(28). (**d**) Joint-acceleration trajectories synthesized by SALCA scheme (24)–(28).

This group of simulative experiments is performed with the tight velocity-layer and acceleration-layer physical limits. The TVLC settings are considered, except for each element in $v^{+}\left(t\right)$ and $a^{+}\left(t\right)$ being, respectively, set as $1.1-0.25\sin^{2}\left(t/2\right)$ rad/s and $8-0.25\sin^{2}\left(t\right)$ rad/s${\;\;}^{2}$. The initial configuration ${\;\;}^{3}\theta \left(0\right)={\;\;}^{2}\theta \left(0\right)$ and the desired configuration ${\;\;}^{3}\theta_{d}={\;\;}^{2}\theta_{d}$. The simulative results synthesized by the EALCA scheme (19)–(23) are shown in Figure 8. From Figure 8a,b, one sees that the four-link planar arm equipped with the EALCA scheme (19)–(23) completes the task with all physical limits satisfied. The corresponding configuration difference is $\epsilon ={\left[3.51612,2.13871,1.36877,0.213871\right]}^{T}\times 10^{-8}$ rad. To verify the effectiveness of the SALCA scheme (24)–(28), the TILC settings are considered except for $v^{+}$ and $a^{+}$ being, respectively, set as 1 rad/s and 3.5 rad/s${\;\;}^{2}$. The simulative results are displayed in Figure 8c,d. From the figure, one sees that the four-link planar arm equipped with the SALCA scheme (24)–(28) also completes the task with all physical limits satisfied. In addition, the corresponding configuration difference is $\epsilon ={\left[6.81465,2.48578,1.34053,0.206452\right]}^{T}\times 10^{-8}$ rad.

To sum up, in those three groups of simulative experiments, the four-link planar arm equipped with the proposed acceleration-layer CA schemes all successfully complete the CA task with all physical limits satisfied, which verifies the efficiency and effectiveness of the proposed CA schemes.

### 5.2. Application to UR3 Spatial Arm

In this subsection, three group experiments based on the UR3 spatial arm are conducted and their results are presented.

#### 5.2.1. Experiment Group 4

As a typical redundant robot arm, the UR3 spatial arm has six DOF and works in three-dimensional space. In this subsection, the UR3 spatial arm is simulated to complete the CA for experimental verification. The Denavit–Hartenberg (D–H) parameters of the UR3 spatial arm are presented in Table 1.

This group of simulative experiments is performed in a loose-constraint case. For clarity, the TVLC settings and TILC settings are defined first. When the EALCA scheme (19)–(23) is applied, the TVLC settings for the UR3 spatial arm are investigated: each element in $p^{\pm }\left(t\right)$, $v^{\pm }\left(t\right)$, and $a^{\pm }\left(t\right)\in R^{6}$ is $\pm 15\mp 0.25\sin^{2}\left(t/2\right)$ rad, $\pm 15\mp 0.25\sin^{2}\left(t/2\right)$ rad/s, and $\pm 15\mp 0.25\sin^{2}t$ rad/s${\;\;}^{2}$, respectively. When the SALCA scheme (24)–(28) is applied, the TILC settings for the UR3 spatial arm are investigated: $p^{-}={\left[-\pi /2,-\pi ,-\pi ,-\pi /2,0,-\pi /2\right]}^{T}$ rad and $p^{+}={\left[\pi /2,0,0,\pi /2,\pi ,\pi /2\right]}^{T}$ rad; each element in $v^{\pm }$ and $a^{\pm }\in R^{6}$ is ±11 rad/s and ±11 rad/s${\;\;}^{2}$, respectively.

**Figure 9:**
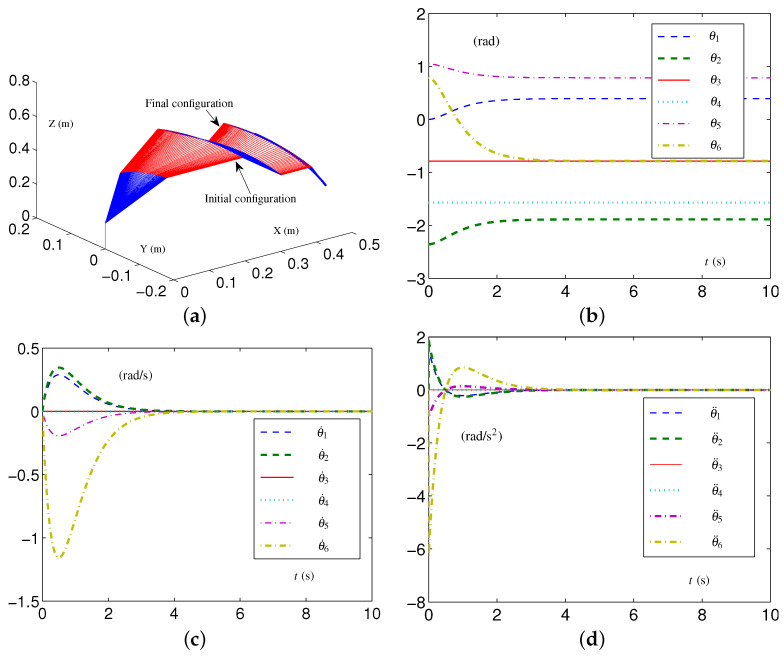
Synthesized results on UR3 spatial arm equipped with EALCA scheme (19)–(23) with loose physical limits satisfied. (**a**) Initial and final configurations. (**b**) Joint-angle trajectories. (**c**) Joint-velocity trajectories. (**d**) Joint-acceleration trajectories.

The initial configuration ${\;\;}^{4}\theta \left(0\right)$ is set as ${\left[0,-3\pi /4,-\pi /4,-\pi /2,\pi /3,\pi /4\right]}^{T}$ rad, and the desired configuration is set as ${\;\;}^{4}\theta_{d}={\left[\pi /8,-3\pi /5,-\pi /4,-\pi /2,\pi /4,-\pi /4\right]}^{T}$ rad. The simulative results synthesized by the EALCA scheme (19)–(23), with the TVLC settings for the UR3 spatial arm, are presented in Figure 9. It can be seen from Figure 9a that the CA task is performed very well, and one sees that all joint variables satisfy the physical limits during the CA task. The configuration difference $\epsilon ={\left[2.12483,2.52508,0,0,1.41277,8.33233\right]}^{T}\times 10^{-8}$ rad is obtained. In addition, the UR3 spatial arm equipped with the SALCA scheme (24)–(28), with the TILC settings for the UR3 spatial arm, also completes the CA task well; these results are omitted due to the limitations of this study.

#### 5.2.2. Experiment Group 5

In this group of experiments, the UR3 spatial arm equipped with the proposed CA scheme performs the CA task with tight velocity-layer and acceleration-layer physical limits, similar to Experiment Group 3. The initial configuration ${\;\;}^{5}\theta \left(0\right)={\;\;}^{4}\theta \left(0\right)$ and desired configuration ${\;\;}^{5}\theta_{d}={\;\;}^{4}\theta_{d}$. When the EALCA scheme (19)–(23) is applied, the TVLC settings for the UR3 spatial arm are set except for each element in $v^{-}\left(t\right)$ and $a^{-}\left(t\right)\in R^{6}$ being $-1.1+0.25\sin^{2}\left(t/2\right)$ rad/s and $-2+0.25\sin^{2}t$ rad/s${\;\;}^{2}$, respectively. When the SALCA scheme (24)–(28) is applied, the TILC settings for the UR3 spatial arm are set except for each element in $v^{-}$ and $a^{-}\in R^{6}$ being set as −1.1 rad/s and −2 rad/s${\;\;}^{2}$, respectively.

**Figure 10:**
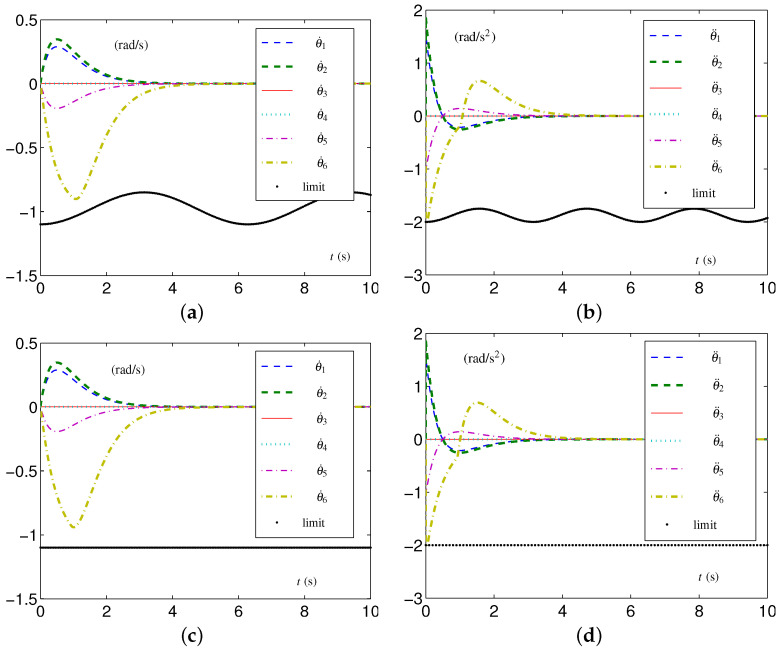
Synthesized results on UR3 spatial arm with tight velocity-layer and acceleration-layer physical limits satisfied. (**a**) Joint-velocity trajectories by EALCA scheme (19)–(23). (**b**) Joint-acceleration trajectories by EALCA scheme (19)–(23). (**c**) Joint-velocity trajectories by SALCA scheme (24)–(28). (**d**) Joint-acceleration trajectories by SALCA scheme (24)–(28).

The simulative results of joint velocities and joint accelerations synthesized by the EALCA scheme (19)–(23) are presented in Figure 10a,b. The corresponding configuration difference $\epsilon ={\left[1.92117,2.30540,0,0,1.28078,1.63878\right]}^{T}\times 10^{-8}$ rad is obtained. Figure 10c,d describe the results synthesized by the SALCA scheme (24)–(28) and display the trajectories of joint velocities and joint accelerations, respectively. The configuration difference of $\epsilon ={\left[1.75767,2.10921,0,0,1.17178,1.62892\right]}^{T}\times 10^{-8}$ rad is obtained.

In comparing the results with those synthesized with the TVLC settings for the UR3 spatial arm shown in Figure 9, we evidently see that the value of ${\dot{\theta }}_{6}$ in Figure 9c is smaller than −1.1 rad/s at near 0.5 s, while the values of ${\dot{\theta }}_{6}$ in Figure 10a,c are kept within velocity-layer constraints. In addition, the value of ${\dot{\theta }}_{6}$ in Figure 10d is smaller than −2 rad/s at the beginning of the experiment, while the values of ${\dot{\theta }}_{6}$ in Figure 10b,d are kept within acceleration-layer constraints. The configuration differences do not increase even if the UR3 spatial arm has tight multilayer constraints. All configuration difference are of order −8, as are those synthesized with loose physical limits. The results shown in Figure 10 verify the effectiveness of the proposed acceleration-layer CA schemes under those conditions.

#### 5.2.3. Experiment Group 6

To further verify the effectiveness of the proposed CA schemes, we conduct a sixth group of simulative experiments with the UR3 spatial arm equipped with the proposed CA schemes and conventional CA scheme. Again, we exchange the initial configuration and the desired configuration in Experiment Group 5, i.e., the initial configuration ${\;\;}^{6}\theta \left(0\right)={\;\;}^{5}\theta_{d}$, and the desired joint-angle vector is set as ${\;\;}^{6}\theta_{d}={\;\;}^{5}\theta \left(0\right)$.

When the EALCA scheme (19)–(23) is applied, the TVLC settings for the UR3 spatial arm are set except for each element in $v^{+}\left(t\right)$ and $a^{+}\left(t\right)\in R^{6}$ being $1.1-0.25\sin^{2}\left(t/2\right)$ rad/s and $2-0.25\sin^{2}t$ rad/s${\;\;}^{2}$, respectively. When either the SALCA scheme (24)–(28) or CALCA scheme (29)–(33) is applied, the TILC settings for the UR3 spatial arm are set except for each element in $v^{+}$ and $a^{+}\in R^{6}$ being set as 1.1 rad/s and 2 rad/s${\;\;}^{2}$, respectively.

Comparative simulative experiments with tight velocity-layer and acceleration-layer physical limits are performed. All tasks are effectively completed with physical limits satisfied. Due to space limitations, the simulative results in the form of a figure are omitted here, while the configuration differences are displayed in Table 2. From the data in the table, three schemes are generally effective, the accuracy of task completion is basically consistent, and the SALCA scheme (24)–(28) has comparative advantages.

In summary, three groups of simulative experiments are performed based on the UR3 spatial arms equipped with the proposed CA schemes. The constraints in Experiment Group 4 are loose, while the constraints in Experiment Group 5 and Experiment Group 6 are tight at the velocity layer and acceleration layer, exchanging the initial configuration and desired configuration. The results show that the proposed CA schemes have superiority compared with the conventional CA scheme.

### 5.3. Physical Experiments

This section presents the application of the EALCA scheme (19)–(23) and SALCA scheme (24)–(28) to a real Kinova Jaco2. The Kinova Jaco2 arm has six DOF, and its angle limits are obtained as $p^{+}={\left[174.5328,5.4105,5.9516,174.5328,174.5328,174.5328\right]}^{T}$ rad and $-p^{-}=[-174.5328,0.8727,0.3316,-174.5328,$${-174.5328,-174.5328]}^{T}$ rad. Two simulations are performed first based on a simulated Kinova Jaco2 arm to guarantee experiment safety. This arm equipped with the SALCA scheme (24)–(28) wants to perform the CA task having tight velocity-layer and acceleration-layer physical limits. The corresponding constraints, as well as the initial configuration ${\;\;}^{7}\theta \left(0\right)$ and desired configuration ${\;\;}^{7}\theta_{d}$, are given as below.

$$\begin{array}{c} {\;\;}^{7}\theta \left(0\right)={\left[-4.2460,2.0192,2.8408,3.8620,-1.9356,3.4888\right]}^{T}\;\mathrm{rad},\;\;\;\;\;\;\;\\ {{\;\;}^{7}\theta }_{d}={\left[-4.9670,2.0672,3.4198,4.2950,-2.3206,3.0228\right]}^{T}\;\mathrm{rad},\;\;\;\;\;\;\;\;\;\\ v^{+}=-v^{-}={\left[2.0,2.0,2.0,2.0,2.0,2.0\right]}^{T}\;\mathrm{rad}/s,\;\;\;\;\;\;\;\;\;\;\;\;\;\;\;\;\;\;\;\;\;\;\\ a^{+}=-a^{-}={\left[0.5,0.5,0.5,0.5,0.5,0.5\right]}^{T}\;\mathrm{rad}/s^{2}.\;\;\;\;\;\;\;\;\;\;\;\;\;\;\;\;\;\;\;\;\; \end{array}$$

**Figure 11:**
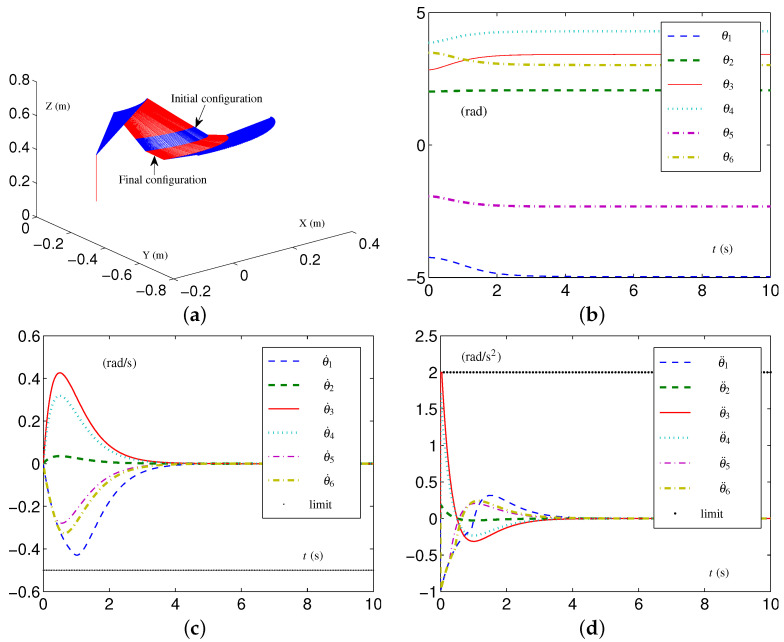
Synthesized results on Kinova Jaco2 arm by SALCA scheme (24)–(28) with tight velocity- and acceleration-layer physical limits satisfied. (**a**) Initial and final configurations. (**b**) Joint-angle trajectories. (**c**) Joint-velocity trajectories. (**d**) Joint-acceleration trajectories.

The corresponding results, which are presented in Figure 11, indicate that the joint configuration is adjusted automatically and successfully by the SALCA scheme (24)–(28). Specifically, Figure 11c shows that each joint velocity value remains within their limited region [−0.5,0.5] m, and Figure 11d shows that each joint acceleration value remains within their limited region [−2,2] m.

**Figure 12:**
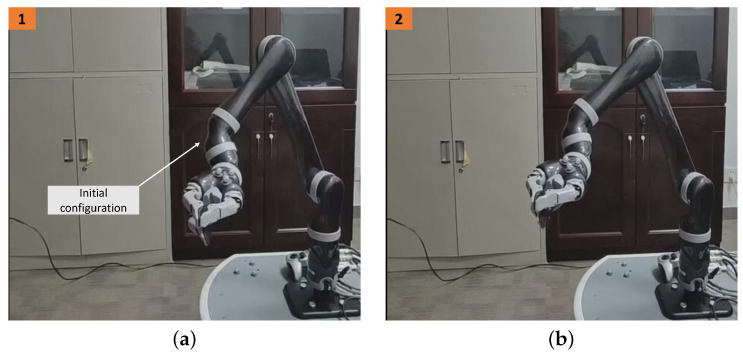

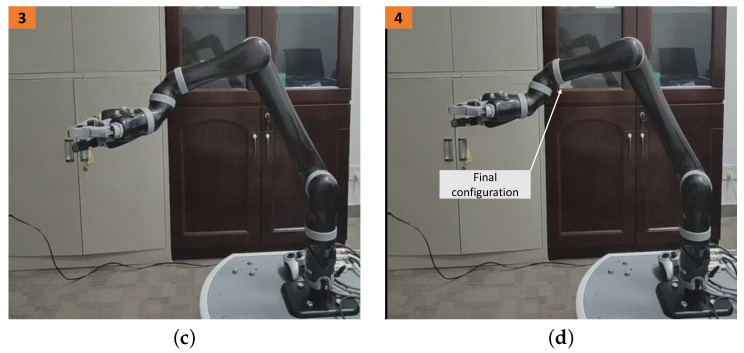
Experimental results obtained by EALCA scheme (19)–(23) with tight velocity- and acceleration-layer physical limits satisfied.

The physical experiment on the Kinova Jaco2 arm equipped with the SALCA scheme (24)–(28) is performed, and the results are displayed in Figure 12. The snapshots in Figure 12a,d, respectively, show the initial and final configurations of the arm, and the snapshots in Figure 12b,c are the process pictures of the CA. The arm successfully completes the CA task. Additionally, the EALCA scheme (19)–(23) is also investigated based on the Kinova Jaco2 arm, and the CA task is also completed successfully. The experimental results are similar, so they are not presented in this study. In summary, the physical experiments of the CA further verify the effectiveness and practicability of the proposed CA schemes.

## 6. Conclusions

In this study, we proposed two acceleration-layer CA schemes via the ZNE method, and one of them improved the CA scheme of redundant robot arms while satisfying three-layer time-variant physical limits. The simulative experiments comparing the conventional scheme with the proposed acceleration-layer CA schemes were conducted based on a four-link planar arm, a six-DOF UR3 spatial arm, and a Kinova Jaco2 arm. The experimental results showed the correctness, effectiveness, and superiority of the proposed CA schemes. We will further study the object-oriented modeling of manipulative robots and realize the efficient control of robots in the future. In addition, combining the position and orientation of the end effector to study the control of redundant robot arms will be a future research direction.

## Figures and Tables

**Table 1:** D–H parameters of UR3 spatial arm.

Joint	$\theta_{i}$	$d_{i}$ (m)	$a_{i}$ (m)	$\alpha_{i}$ (rad)
1	$\theta_{1}$	0.1519	0	π/2
2	$\theta_{2}$	0	−0.2437	0
3	$\theta_{3}$	0	−0.2133	0
4	$\theta_{4}$	0.1124	0	π/2
5	$\theta_{5}$	0.0854	0	−π/2
6	$\theta_{6}$	0.0819	0	0

**Table 2:** Configuration differences obtained by UR3 spatial arm with tight velocity-layer and acceleration-layer constraints in Experiment Group 6.

ϵ	EALCA Scheme (19)–(23)	SALCA Scheme (24)–(28)	CALCA Scheme (29)–(33)
$\epsilon_{1}$	$1.74721\times 10^{-8}$	$1.56053\times 10^{-8}$	$1.70739\times 10^{-8}$
$\epsilon_{2}$	$2.09665\times 10^{-8}$	$1.87264\times 10^{-8}$	$2.04886\times 10^{-8}$
$\epsilon_{3}$	0	0	0
$\epsilon_{4}$	0	0	0.0145794
$\epsilon_{5}$	$1.16481\times 10^{-8}$	$1.04035\times 10^{-8}$	$1.13826\times 10^{-8}$
$\epsilon_{6}$	$1.62012\times 10^{-7}$	$1.33826\times 10^{-7}$	$1.05391\times 10^{-7}$

## Data Availability

The data presented in this study are available on request from the corresponding author.
